# Residual Stress Build-Up in Aluminum Parts Fabricated with SLM Technology Using the Bridge Curvature Method

**DOI:** 10.3390/ma15176057

**Published:** 2022-09-01

**Authors:** Quoc-Phu Ma, Jakub Mesicek, Frantisek Fojtik, Jiri Hajnys, Pavel Krpec, Marek Pagac, Jana Petru

**Affiliations:** 1 Department of Machining, Assembly and Engineering Metrology, Faculty of Mechanical Engineering, VSB-Technical University of Ostrava, 70833 Ostrava, Czech Republic; 2 Department of Applied Mechanics, Faculty of Mechanical Engineering, VSB-Technical University of Ostrava, 70833 Ostrava, Czech Republic; 3 V-NASS, A.S., Halasova 2938/1a, 70300 Ostrava, Czech Republic

**Keywords:** selective laser melting (SLM), residual stress, hole drilling method (HDM), bridge curvature method (BCM), finite element analysis (FEA), aluminum alloys

## Abstract

In metal 3D printing with Selective Laser Melting (SLM) technology, due to large thermal gradients, the residual stress (RS) distribution is complicated to predict and control. RS can distort the shape of the components, causing severe failures in fabrication or functionality. Thus, several research papers have attempted to quantify the RS by designing geometries that distort in a predictable manner, including the Bridge Curvature Method (BCM). Being different from the existing literature, this paper provides a new perspective of the RS build-up in aluminum parts produced with SLM using a combination of experiments and simulations. In particular, the bridge samples are printed with AlSi10Mg, of which the printing process and the RS distribution are experimentally assessed with the Hole Drilling Method (HDM) and simulated using ANSYS and Simufact Additive. Subsequently, on the basis of the findings, suggestions for improvements to the BCM are made. Throughout the assessment of BCM, readers can gain insights on how RS is built-up in metallic 3D-printed components, some available tools, and their suitability for RS prediction. These are essential for practitioners to improve the precision and functionality of SLM parts should any post-subtractive or additive manufacturing processes be employed.

## 1. Introduction

The complex nature of residual stress (RS) in parts printed with Selective Laser Melting (SLM) technology is caused by the interaction between the mechanical, thermal, and metallurgical properties of the metals [1]. This stress is self-equilibrium within an elastic component regardless of the external load application, which can either improve or degrade the component strength during operation. By definition, there are three types of RS (type I, type II, and type III) that exist within a body and are classified based on the scale of stress evaluation [1,2]. Particularly, type I is macroscopic and homogeneous within many grains of the material. Type I is caused by the production technology, assembly, operation, transportation, and application of variable loads during testing, etc. Type II, on the other hand, is microscopic and intergranular. It is caused by the inhomogeneous longitudinal thermal expansion of the grains during the phase-changing process. Last, type III is submicroscopic and is associated with defects on the subgrain level. RS measurement methods are classified as destructive, non-destructive, and semi-destructive based on their principles and the type of RS to be assessed, as summarized in [3].

The formation of RS formation in parts produced with SLM shares several similarities with welded parts, however, with a more complex nature due to three distinct characteristics of its thermal cycle [4]: (1) intensive heating creating large temperature gradients, (2) fast-paced cooling and solidifying due to the small volume of the melt pool, and (3) melting of a new consecutive layer while remelting the former solidified layer. The formation of RS in the SLM process was originally described in [5], whose illustration was corrected in [4]. Being different from welding, the formation of RS in the SLM process is considerably more sophisticated, as rapidly heated and cooled layers are stacked, which could lead to print abortion due to cracking during printing. In addition, after the components are removed from the base plate, RS is redistributed throughout their bodies, resulting in shape distortion that could deteriorate their functionality [4]. This predictable behavior has been used as a simplified approach to quantify RS in SLM components, specifically with two geometries, the bridge and the cantilever, as reviewed in [6]. The two were designed so that they allow geometric distortion, or stress release, solely in one direction, thus isolating the effect of the RS to be assessed. Specifically, the bridge-shaped geometry was first described in [7], whose piers curl up after being cut from the base plate and the angle between the two bottom planes of the piers is measured for RS evaluation. Due to the undemanding requirements in terms of material usage, printing time, and the pre- and post-process, as well as angle measurement, the Bridge Curvature Method (BCM) has been widely accepted by the research community as a rapid means to study RS [8,9,10]. Additionally, as claimed in [6], the measured angle can then be imported into Finite Element Analysis (FEA) software as a boundary condition to reversely calculate the releasable RS in the bridge before cutting. This approach is similar to how the authors in [11] used the results of the contour method to back-calculate the RS distribution in a component.

There are two FEA methods that are often used to evaluate the shape distortion and RS of 3D-printed components, i.e., the thermomechanical method and the inherent strain method. The former is conventionally used to calculate welding distortion and has been used extensively in 3D-printing simulations by the research community, as reviewed in [12], and for simple geometries, as in [13,14,15,16]. Fundamentally, this approach simulates the moving heat source and couples the thermal and linear/non-linear properties of the materials, making it computationally demanding and sensitive to thermal–mechanical experimental measurement. In contrast, the latter is more efficient in terms of time and efforts spent on computing and experiments because it only considers the inherent strain of the materials, which is purely mechanical. Specifically, this method assumes that the size of the laser seam is negligible compared to the size of the in-built component, and the thermomechanical history of the laser seams are insignificantly different from one another. Therefore, only the final geometric distortion of the printed component is needed as input. Thanks to the advantages that the method offers, it is more suitable for the simulation of large and complex components in the industry and has been widely adapted in [17,18,19,20].

Inspired by the above studies, this paper investigates the accumulation of RS in 3D-printed metal specimens by means of simulation and physical tests on the macroscopic level. Specifically, the bridge samples, designed following the BCM, are 3D printed with AlSi10Mg and investigated. AlSi10Mg was chosen for the study because it is a widely-used material, besides stainless steel, for printing. The two commercial FEA software, namely ANSYS and Simufact Additive, are deployed for the numerical study. We use ANSYS software for conventional elastic simulations with distorted angles as boundary conditions to reversely calculate the elastic stress distribution of the bridges. Additionally, Simufact Additive is used to simulate the printing process of the bridges and their corresponding stress distributions. Bridge samples are divided into two packs for RS examination; one is kept as-built and the other is heat treated at 170 °C for 6 h. The RS of all the bridges is examined with the Hole Drilling Method (HDM) according to the ASTM E837-20 standard and subsequently compared with the simulation results. It should be noted that the principles for the physical measurement and simulation used herein are the same as those for different metallic materials, as long as the material properties are known.

## 2. Materials and Methods

### 2.1. Sample Design and Fabrication

**Figure 1:**
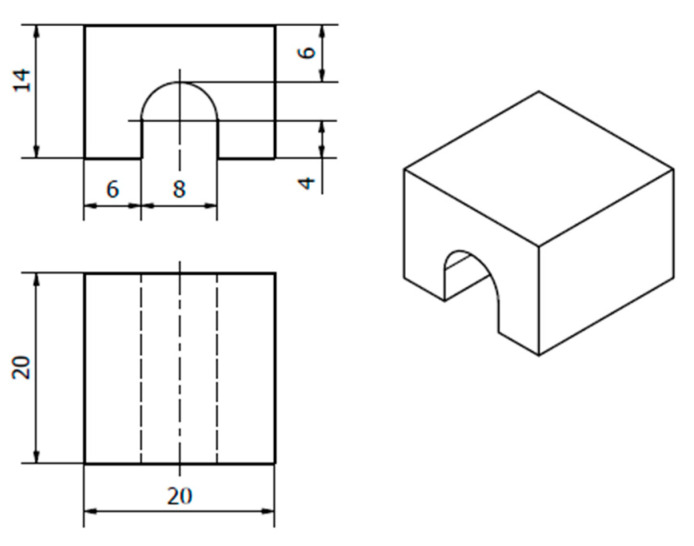
Bridge geometry and dimensions in mm.

Unlike the small geometry of the bridge in the original article [7], we redesigned the bridge to approximately twice its size, aiming for a larger distorted angle at the bottom and a larger top surface to facilitate HDM measurement and RS evaluation. The redesigned bridge is shown in Figure 1.

**Figure 2:**
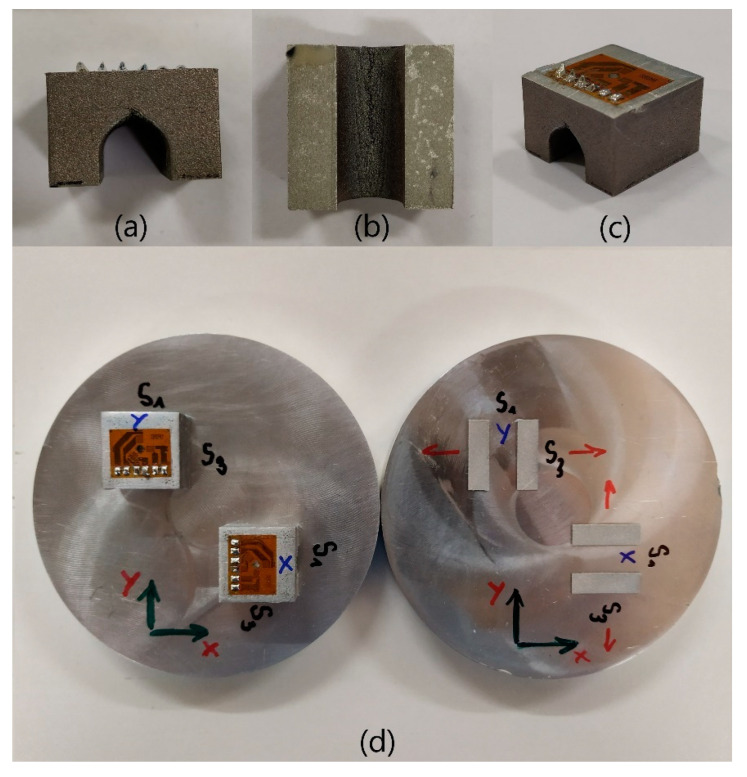
Bridges for HDM measurements: (**a**) front view, (**b**) bottom view, (**c**) isometric view, and (**d**) bridge with notation, before cutting (**left**) and after cutting (**right**).

Trumpf Truprint 1000 was used to print 8 bridges in total on 4 base plates (2 bridges on each base plate). The material for the bridges was AlSi10Mg, which had been recycled 30 times. This was done because it is common practice to use recycled powder for printing. To investigate the effect of printing direction on the RS, we oriented the bridges in the Ox and Oy directions, whereas Oz is the printing direction. Ox is the direction of movement of the powder recoater, and Oy is normal to the machine door. The bridges printed in these Ox and Oy were denoted as X and Y bridges. This coordinate system is used henceforth for both measurement and simulation, as is shown in Figure 2. For printing, the bridges were rotated 5° clockwise around the Oz axis to avoid full edge-to-edge collision between the bridges and the recoater, which may lead to printing failure. The bridges were printed using the printing parameters as shown in Table 1.

After printing, the 4 base plates were divided into 2 packs for RS assessment. The first pack remained as its as-built condition (pack 0 h) and the second was heat treated at 170 °C for 6 h (pack 6 h). The heat treatment was carried out using the LH120/12 furnace from Nabertherm GmbH. A temperature of 170 °C was chosen because it is often used for the stress relaxation of AlSi10Mg while ensuring that there are no microstructural changes [21,22]. Furthermore, an internal study comparing the tensile properties of AlSi10Mg undergoing heat treatments of 170 °C from 0–7 h proved that the pack that was aged for 6 h had the best material properties. The comparison of the material properties of 0 h and 6 h is shown in Table 2.

### 2.2. Measurement

For the comparison of RS stress, a base plate was separated from each pack (0-h pack and 6-h pack) and the bridges were cut from them to measure the distorted angles with an Alicona Infinite Focus 5G optical microscope from Alicona Imaging GmbH. Subsequently, all the bridges were subjected to RS assessment with HDM (both the ones that remained on the base plates and the cut-off ones). First, referring to [23,24] for HDM measurement, the top surface of the bridge was grinded to obtain a sufficient roughness to glue the rectangular strain gauge rosette. The surface was treated with fine hand grinding and etching so that no additional RS was introduced. In the middle of the strain gauge, a 2 mm diameter hole was gradually drilled to 1 mm deep with a step of 0.05 mm. The measurement and evaluation of the results were carried out according to the ASTM E 837-20 standard. The evaluation was carried out under the assumption of uniformly-distributed RS. Due to the fact that the RS does not vary much within each 0.1 mm depth increment, only stress values per 0.1 mm (instead of 0.05 mm) were recorded and plotted to compare with the simulation for a clearer data report. Figure 2 shows the bridges with notation.

The strain gauge rosettes can be seen on the top of the bridges. The in-used coordinate systems correspond to the descriptions in Section 2.1. It can be noticed that the lack of supports in the curvature region results in a crack-like pattern that can be up to approximately 1 mm high. Additionally, as noted on the base plate, for X bridge, S1 and S3 correspond to the normal stress in the Ox and Oy directions, respectively, and vice versa for Y bridge. As previously mentioned, the bridge is designed so that after cutting, only S3 is released in the direction of the red arrows as the piers distort in the same direction, while S1 is not. It should be noted as well that the normal stress distribution in the Oz direction cannot be measured with this experimental setup.

### 2.3. Numerical Study

#### 2.3.1. Material Properties

Tensile specimens were printed with the same printing parameters and subsequently heat-treated together with the bridges in this study. Their mechanical properties are reported in Table 2. Remarkably, for the simulation in Simufact, only the material properties of the 0-h pack were used because it was anticipated that the change in mechanical properties could be captured with the built-in heat treatment function.

#### 2.3.2. Inherent Strain Method

The total residual strain in a welded or 3D-printed component can be expressed in the following equation:

(1) $$\varepsilon_{total}=\varepsilon_{e}+\varepsilon_{p}+\varepsilon_{th}+\varepsilon_{ph},$$

where there are the elastic strain ($\varepsilon_{e}$), the plastic strain ($\varepsilon_{p}$), the thermal strain ($\varepsilon_{th}$), and the strain induced from the phase transition ($\varepsilon_{ph}$). Subsequently, the inherent strain is defined as follows:

(2) $$\varepsilon^{*}=\varepsilon_{total}-\varepsilon_{e},$$

The inherent strain governs the complete history of the printing process ($\varepsilon_{p}$, $\varepsilon_{th}$, $\varepsilon_{ph}$) and solely reflects it with the final geometric distortion, after the part is cut out of the base plate and the elastic strain ($\varepsilon_{e}$) is released [25]. Furthermore, it should be noted that the $\varepsilon_{total}$ is compatible and the $\varepsilon^{*}$ is incompatible, as explained and illustrated in [26]. Due to its definition, the inherent strain method is able to remarkably simplify the simulation process while delivering a considerably accurate RS prediction.

In ANSYS, after the distorted angle of the bridge is obtained from the physical measurement, it is applied to the unstressed bridge model to reversely calculate the elastic normal stress that is released during cutting. Referring to Equation (2), it is equal to $\varepsilon^{*}=\varepsilon_{total}-\varepsilon_{e}$, or in words, the total RS in the bridge before cutting subtracts the one after cutting.

For the RS results in Simufact Additive, the inherent strain method is used. Specifically, $\varepsilon^{*}$ is decomposed into three directions, being $\varepsilon_{xx}$, $\varepsilon_{yy}$, and $\varepsilon_{zz}$, which can be calculated by means of cantilevers through a process called calibration [27]. First, the cantilevers are printed with a specific material and a set of printing parameters. Then, they are cut out so that the elastic strain can be released, resulting in shape distortion. The displacements at the tip of the cantilevers are measured and entered into Simufact Additive for the inherent strain calculation. After calibration, a set of three-directional inherent strains is obtained, which can be used later for the simulation of other components.

**Figure 3:**
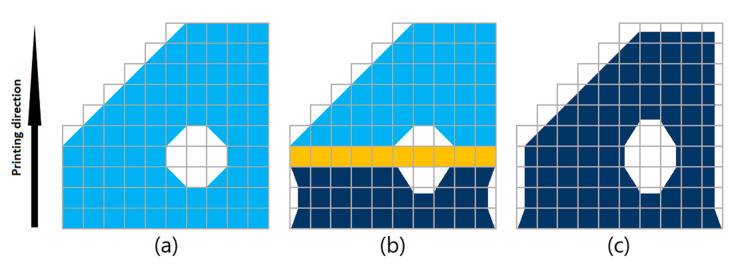
From left to right: (**a**) Geometry approximated with voxel (light blue), (**b**) consecutive activation (orange) and application of inherent strain (making part of the printed component shrink in size, navy blue), and (**c**) completely printed component.

Regarding the printing simulation, Simufact Additive uses the deactivate/activate approach to describe the inherent strain effect, which is illustrated in Figure 3.

Figure 3 helps to explain the simulation in 2D, which can be extended to the 3D case. First, the geometry is approximated with voxels (hexahedron elements), which are placed layer by layer on top of each other. Each voxel is filled with material and assigned a volume fraction (with a 100% volume fraction being the voxel that is fully filled with material). Stiffness is scaled directly with the volume fraction, which is equal to the percentage of material filled in a voxel volume. The collection of voxels representing the geometry is created first in an unactive state. Then, layer upon layer of voxels are consecutively activated and assigned with the calibrated inherent strain values, making them shrink in size. This shrinking effect is calibrated so that it is closest to reality using the calibration process mentioned previously. The shrinkage is summed throughout the layers to yield the final distorted shape of the printed geometry. In particular, if the material or the printing parameters used to print the components change, the calibration must be performed again to obtain a new set of inherent strains.

It should be noted that the simulation approaches used in this study do not employ any force or temperature as boundary conditions, but only the final distorted angles (for ANSYS) and inherent strains (for Simufact Additive). Moreover, the two methods are purely mechanical and are based solely on the final distorted shape of the printed parts. Thus, they serve as quick ways to quantify the RS inside the printed parts without the need for any information related to laser path, laser speed, printing strategy, etc.

#### 2.3.3. ANSYS Simulation Setup

As introduced in advance, the BCM is a simplified approach to quantify the RS within the 3D-printed components. After being removed from the base plate, the distorted angle can be utilized as a boundary condition to reversely calculate the stress distribution on the bridge with ANSYS Workbench 2019 R3. To reduce the computational time, half of the bridge was used and designed in the default add-in, Design Modeler. The symmetry boundary condition was applied to the mirror plane of the bridge geometry. The geometry was meshed with linear hex elements of up to 0.4 mm in size, resulting in 35,100 elements and 151,119 nodes. A visual probe was created at the middle point on the top plane of the bridge with a depth of 1 mm to measure the RS. The material models used were 0 h and 6 h from Table 2.

#### 2.3.4. Simufact Additive Simulation Setup

The material properties for simulation in Simufact Additive were edited from the one available in the database, namely AlSi10Mg_powder, with tested data of the 0-h pack from Table 2. Additionally, the flow curves were re-scaled using yield strength, ultimate strength, and ductility. The calibration was carried out with cantilevers printed from the printing parameters shown in Table 1, and the set of inherent strains that was obtained is $\varepsilon_{xx}$ = −0.00375, $\varepsilon_{yy}$ = −0.00337, and $\varepsilon_{zz}$ = −0.03. Notably, the minus sign indicates the shrinkage of the components. Additionally, the bridges were meshed with a voxel size of H0.5 × W0.5 × L0.5 mm. There are 223,768 voxels, 258,179 nodes, and 70 layers. Each node has three translational degrees-of-freedom (DOF). As for the simulation process, there are two to three consecutive stages, i.e., build, (heat treatment), and immediate release. For post-processing, interpolation between two nodes of a voxel was done to obtain the stresses, which are 0.1 mm apart and correspond to the measurement with HDM.

## 3. Results and Discussion

### 3.1. Distorted Angles of the Bridges

Table 3 shows the distorted angles of the X and Y bridges taken from the 0-h and 6-h packs.

The distorted angles are relatively small and, therefore, are not visible in Figure 2a–c. The angles were halved and inserted into the symmetric bridge models in ANSYS as the boundary condition for the reverse stress calculation. It can be noticed that the 6-h heat treatment can reduce the distorted angles (or the RS) on the X and Y bridges to approximately 50%.

### 3.2. Stress Distribution Results in ANSYS

**Figure 4:**
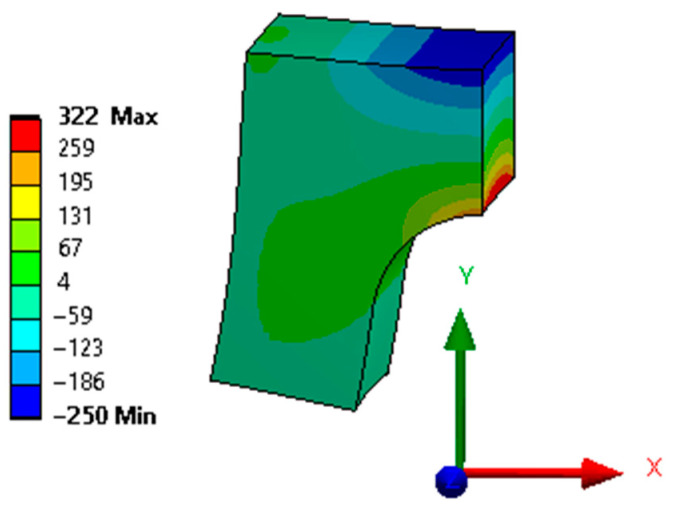
Normal stress distribution on the cut bridges calculated with ANSYS in MPa.

Figure 4 shows the reverse stress calculation in ANSYS.

This result is equivalent to the released S3 of the X bridge, which is elastic and in the reverse direction. Following the setup of the studies, there are four ANSYS results in total, being that S3 was released in the X and Y bridges for the 0-h and 6-h packs. The stress distribution of the four results is the same as the one presented in Figure 4, and only the numerical results are different. Thus, only one figure was chosen for illustration, whereas accurate numbers are reported in the following sections.

### 3.3. Stress Distribution Results in Simufact Additive

In Simufact Additive, there are two result types, namely a surface and voxel result. The surface result better represents the geometry of the models; however, it cannot show the internal stress distribution. Therefore, it is recommended that the model be volume-meshed with voxels, and clipping should be performed on the voxel result instead. Technically, the results of the voxels are mapped onto the surfaces for the surface results, which serve as a basis for the stress assessment henceforth. This notation should be taken into account as the reader attempts to replicate the simulation. There is no special technique for mapping or interpolating the numerical results obtained from the simulations.

**Figure 5:**
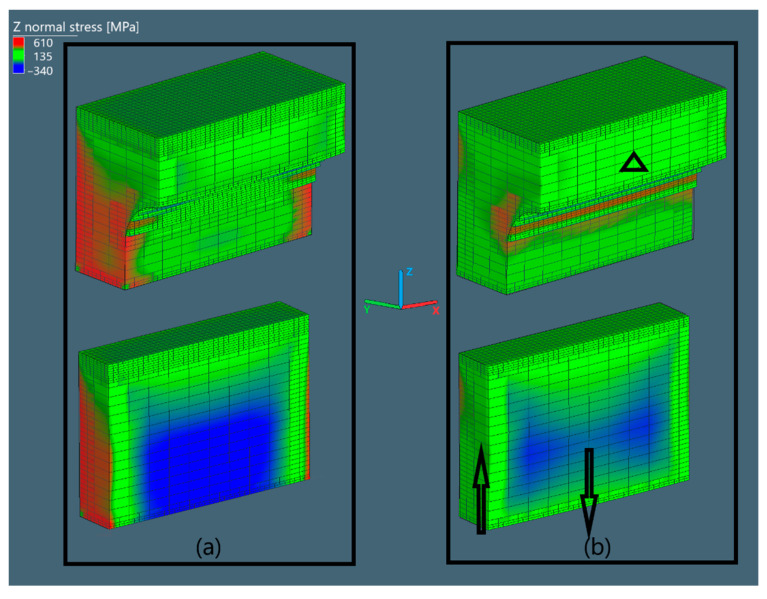
Normal stress in Oz direction of the bridges in Simufact Additive (**a**) before cutting and (**b**) after cutting.

**Figure 6:**
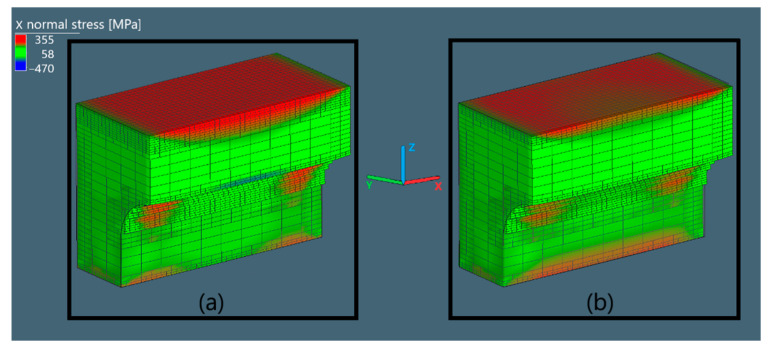
Normal stress in Ox direction of the bridges in Simufact Additive (**a**) before cutting and (**b**) after cutting.

**Figure 7:**
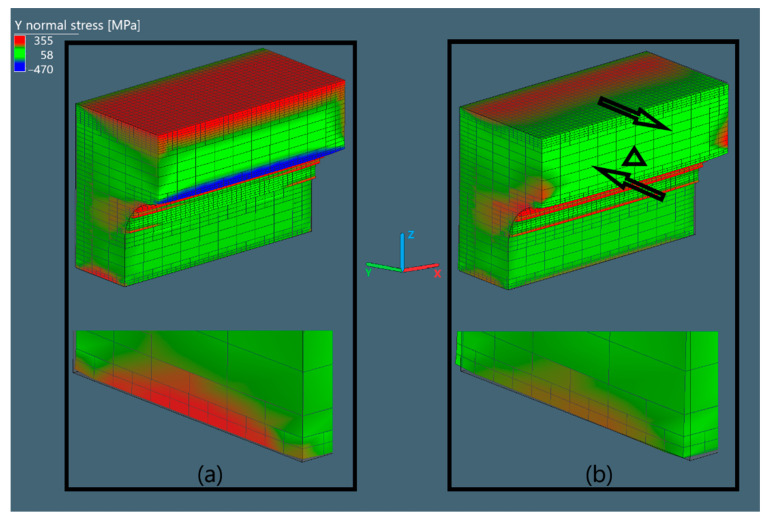
Normal stress in Oy direction of the bridges in Simufact Additive (**a**) before cutting and (**b**) after cutting.

In the Figure 5, Figure 6 and Figure 7 below, the stress distribution on the X bridge is shown. It should be noted that the normal stress distribution in the Ox and Oy directions of the Y bridge is reversed from the X one. However, as denoted in Figure 2, S1 and S3 do not depend on choice of the coordinate system, and only S3 of the cut bridges is released. The normal stress distribution in the Oz direction is similar in terms of magnitude and direction. Indeed, for the simulation results in Simufact Additive, the difference in normal stress levels between the X and Y bridges is less than 1 MPa, which is negligible. Thus, only the results of the X-bridge are shown in this subsection. Moreover, it has to be kept in mind that in the figures, there is an imaginary pivot (marked with triangle) on the symmetric plane of the bridge about which the piers rotate when the RS is released, and direction of the rotation (marked with arrows).

In practice, the stress results in the three directions vary slightly compared to each other because the bridges are located at different positions on the base plate. This principle is called the partial distribution of strain and is expected to be severe for a large base plate, which is not applicable for the base plate size of D100 mm in this study. Additionally, the geometry of the bridge was compared to its undeformed shape (CAD), as represented by the grey transparent box wrapping around the bridge, which can be observed in the close-up view in Figure 7. The curvature of the bridge is not fully captured because the geometry is approximated with voxels. The cross sections of the bridges are clipped with an advanced setting to show the remaining part of the model with elements for a better stress assessment at their inner nodes.

#### 3.3.1. Normal Stress in Oz Direction

The normal stress in Oz direction is shown in Figure 5.

The bridge is first halved, then the pier is further sectioned to show the internal stress distribution in different regions. The middle curvature region experiences no considerable amount of compressive or tensile stress, while the piers do. As the bridge is removed, the pier will seek its equivalence by deflecting in the direction of the black arrows. This happens in the same manner as on the other pier, since the bridge is symmetric. The black triangle is the reference point around which the pier distorts.

#### 3.3.2. Normal Stress in Ox Direction

The normal stress in Ox direction of the X bridge (S1 of X bridge), corresponding to S1, is shown in Figure 6.

The S1 in the X bridge before and after being cut from the base plate only shows a slight change in the middle region of the top layers. This is because there is barely any deformation allowed in the Ox direction for S1 to be released.

#### 3.3.3. Normal Stress in Oy Direction

The normal stress in Oy direction of the X bridge (S3 of X bridge), corresponding to S3, is shown in Figure 7. 

When examining the thin section, it can be observed that the outermost layer of the curvature peak is subjected to a high level of compressive S3, while the upper layers are dominated by tensile S3. After cutting, the tensile stress on the top layers is significantly reduced, causing the piers to distort in the direction of the arrows. It should be noted that all the figures were scaled up 2 times to better illustrate the pier distortion. From the close-up view, it can be observed that the uncut bridge is smaller in size in comparison with its CAD counterpart because of the shrinkage that occurs during the printing. This was indeed calibrated to be the closest to reality by utilizing the inherent strain method as previously mentioned. Subsequently, the bottom close-up figure illustrates how the bridge deforms as stress is released. The bridge solely allows stress release in the Oz and Oy but not Ox direction due to its specific geometry, which can be observed when we compare the stress distribution between Figure 6 and Figure 7 before and after cutting. Additionally, what can be drawn is that the thin curvature section of the bridge is the main source of distortion after cutting.

#### 3.3.4. Summary of Results

The distorted angles and normal stress results from the simulations and HDM measurements are summarized and compared in this section. As previously mentioned, due to the lack of supports, the curvature region of the bridge was not fully printed, resulting in a crack-like pattern, which can be up to approximately 1 mm high (see Figure 2). With regard to Figure 7, this region stores compressive S3, which plays an important role in the final distortion of the piers of the bridge. Thus, the lack of powder in that region is anticipated to lead to undesirable distortion results.

The results are denoted with a number of symbols to facilitate reporting. For the HDM measurements, the RS are denoted by X_HDM and Y_HDM. As previously mentioned, the difference in normal stress levels between the X and Y bridges in Simufact Additive is insignificant—below 1 MPa. Therefore, they are combined and put under the name, X/Y_SM. Moreover, the ΔS3 denotes the released elastic stress ($\varepsilon_{e}$), which is equal to the Uncut S3 that subtracts the Cut S3, ($\varepsilon_{total}-\varepsilon^{*}$)—as given in Equation (2). When released, this elastic component forms a distorted angle between the bridge piers. These angles were subsequently put into ANSYS to reversely calculate the released elastic stress of the X and Y bridges, which are denoted as X_ANS and Y_ANS. Remarkably, as opposed to the simulation, the RS level at 0 mm deep is not measurable in reality; thus, it is not shown in the figures. Additionally, since the normal stress in the Oz direction cannot be measured using the HDM setup in this study, only the normal stress in the Ox direction (S1) and the normal stress in the Oy direction (S3) are presented.

**Figure 8:**
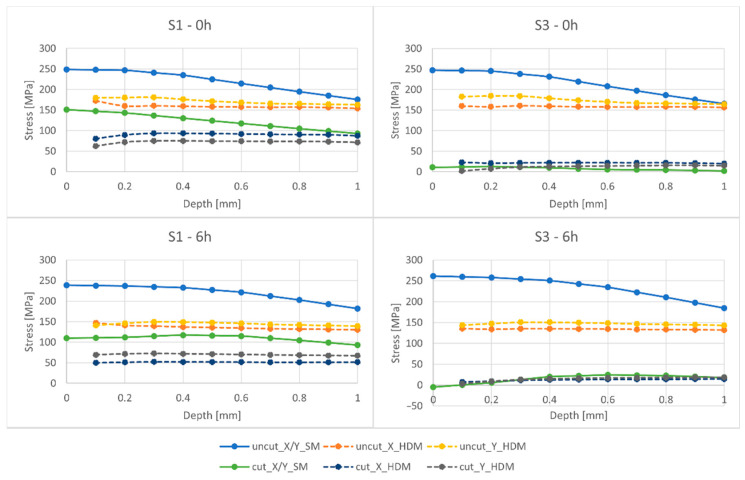
Summary of S1 and S3 results for 0-h and 6-h packs from measurement and simulation.

The normal stress distribution in the X and Y bridges obtained from the simulation and physical tests is compared in Figure 8 (with data from Table A1 and Table A2).

It can be observed that for both the simulation and the HDM measurement, S1 and S3 are below the yield limit of Sy = 279 MPa. This is crucial for HDM measurement, because according to the ASTM standard, theoretically, satisfactory measurement results can be achieved given that the RS does not exceed about 80% of the material’s Sy when HDM is performed on a “thick” material. Additionally, the accuracy of the HDM results also depends greatly on the skill and experience of the user. The authors have sufficient expertise to perform the HDM measurement [28,29,30].

Furthermore, the simulated RS S1 and S3 tend to decrease as the depth increases, while with HDM, the stress remains almost constant within the 1 mm depth. This could be because Simufact Additive assumes that the layers are perfectly bounded together without any porosity in between, which does not hold for the reality. Furthermore, HDM may not capture the stress distribution of the bridges as the drill tip penetrates the stacked layers. Moreover, it can be observed that S1 is partially released after cutting (corresponding to the slighter change in contour plot on the top layers of the bridges in Figure 6), while S3 is entirely released (corresponding to Figure 7). The HDM measurements show that heat treatment for 6 h can decrease the RS, shown by the overall lower level of S1 and S3 in the 6-h bridges before and after cutting in comparison with the 0-h bridges. Remarkably, as AlSi10Mg is an age-hardening material, this heat treatment process can increase the mechanical properties of the bridges as well (see Table 2).

Comparing the S1 and S3 of the 0-h and 6-h bridges, it can be observed that the reduction in RS by heat treatment is not predictable with the inherent strain approach in Simufact Additive, even though the heat treatment process was involved in the printing simulation. The best prediction is S3 for both the 0-h and 6-h packs. This is reasonable because the inherent strains used for the calibration process were obtained from the cantilevers that have been cut when the RS was already released. The RS results before cutting were solely calculated from the final stress state; thus, with great errors. Additionally, the inherent strain method mainly focuses on obtaining the final inherent strain, and then the strain is back-calculated to stress. The implicit way of calculating stress could contribute to the overall error of the RS prediction. Finally, when heat treatment is involved, prediction errors can be greater due to the improper setting of flow curves, which defines the dependence of the strength on the temperature change.

### 3.4. Stress Release of The Bridges

**Figure 9:**
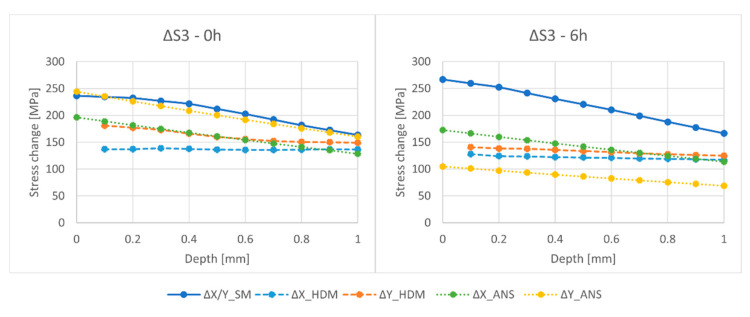
Summary of the ΔS3 results for 0-h and 6-h packs from measurement and simulation.

The normal stress that is released from the X and Y bridges obtained from the simulation and physical tests data is compared in Figure 9 (with data from Table A3 and Table A4). The change in RS is denoted with symbol Δ.

It should be noted that the references for the comparison are the stress release calculated from HDM, ΔX_HDM, and ΔY_HDM. These curves do not start at a depth of 0 mm because it is physically impossible to measure the stress at this depth. The change (release) in stress ΔS3 is better captured in ANSYS in comparison with Simufact Additive. In the reviewed literature, it is appropriate to reversely calculate the elastic RS that is released after cutting using the final geometric distortion of the component as a boundary condition for the FEA simulation. However, this approach is limited to showing only the elastic components of the RS in the components, not the full picture of how the whole RS is distributed in the bridges.

**Figure A1:**
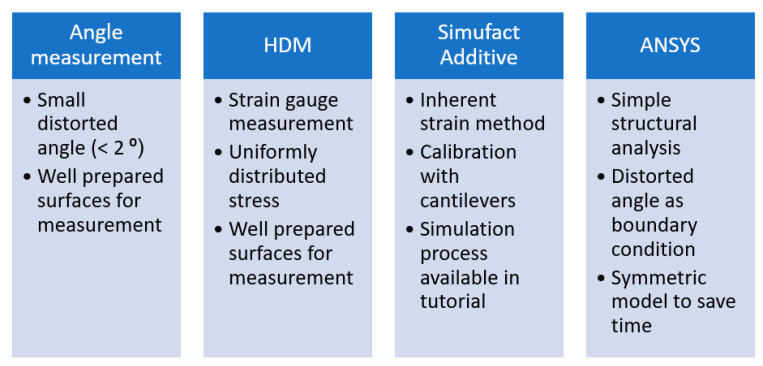
Notes of the four pillows of the study for replication.

From both the simulation and physical measurements, insights for shape distortion and RS prediction are drawn. Specifically, for Simufact Additive, since it approaches the RS prediction problem with the inherent strain method, which is purely mechanical, it is unable to capture the changes of RS with respect to temperature. However, the RS distribution on the printed bridges in Simufact Additive can still serve as references for the physical measurements, that is, for identifying the distortion of the printed parts and critical areas for RS assessment. On the other hand, the classical elastic FEA simulation with angle constraints in ANSYS can predict the released elastic component of the RS relatively well. Nevertheless, it does not provide the whole picture of the RS that exists in the printed components. These insights are applicable for the shape distortion and RS prediction of other components with different geometries as well. Specifically, the inherent strain approach is mostly utilized because it saves computational time and can provide a quick assessment of the shape distortion and RS hot spots. The error between the predicted result and reality can be reduced with a better calculation of the inherent strain (better calibration of Simufact Additive with the real printer), but the results cannot be as accurate as the thermal–mechanical approach. Additionally, similar to how distorted angles were used as a boundary condition in ANSYS, the final shape distortion of any printed part can be used as a constraint in any traditional FEM software to reversely calculate the released RS of that part. The four pillows of this study with key notes are summarized in Figure A1, which should serve as an instruction for later replication.

## 4. Conclusions

By and large, BCM has been accepted among the research community as a quick quantifier for built-in RS. This study reviews and proves the accuracy of some existing methods that can be used to examine bridge geometry. The bridges used in BCM have typical geometric features for 3D-printed parts, i.e., full solid squared column sections printed directly on the base plate (the piers), overhang, and thin sections (the curvature). After understanding how the RS is built-up in different sections of the bridges and how they affect the final shape distortion, readers can relate directly to their metallic 3D-printed components to make appropriate post-printing adjustments (usage of supports, part orientation, design changes, etc.).

To improve the BCM, future work can focus more on setting up the flow curves in Simufact Additive, which is coupled thermal–mechanically, to better correlate the simulation with reality. As for physical tests, other types of RS measurement can be applied to investigate bridges from different points of view. Taking into account the printing of bridges, the number of bridges can be increased so that statistical studies can be carried out. Furthermore, as previously drawn, the thin section is the main source of the distortion of the bridge’s piers. Nonetheless, as we can observe from the printed bridges, the top of the curvature cannot be fully printed because of the lack of supports. This results in the small, crack-like area observed from the front view, which is not filled with material and can be up to 1 mm high. Practically, adding supports for this area is a possible solution to ensure that the curvature is printed successfully. However, supports lead to a smaller amount of RS in the thin section because they provide more sufficient cooling, as heat is subsequently better transferred from the thin section to the base plate. Therefore, future works can also consider changing the geometry of the bridges to make them better RS indicators. In general, given the complex nature of the properties of 3D-printed components, the purely mechanical inherent strain approach in Simufact Additive is more appropriate to rapidly predict the shrinkage and RS distribution of the printed components in the industrial application, and displacement constraints in classical FEA would be better for the back-calculation of the elastic part of the RS.

## Figures and Tables

**Table 1:** Printing parameters.

**Powder**	AlSi10Mg
**Powder particle size**	15–45 µm
**Laser power**	175 W
**Layer thickness**	20 µm
**Focus size**	55 µm
**Printing strategy**	Chessboard (Zig-Zag)
**Laser speed for border/following border/hatching**	2000/1500/1400 mm.s^−1^

**Table 2:** Material properties of the heat-treated packs for 0 h and 6 h.

Time [h]	Young’s Modulus [MPa]	Poisson’s Ratio [-]	Yield Strength [MPa]	Ultimate Strength [MPa]	Ductility [-]
0	60.3	0.31	279	409	0.032
6	75.9	0.31	309	448	0.030

**Table 3:** Measured distorted angles.

	Distorted Angle [°]	
Sample	0 h	6 h
X	1.6	0.7
Y	2.0	1.1

## Data Availability

Data are available in Appendix A.

## Appendix A

**Table A1:** S1 and S3 on the 0 h bridges from Simufact Additive and HDM.

	D [mm]	0.0	0.1	0.2	0.3	0.4	0.5	0.6	0.7	0.8	0.9	1.0
Uncut S1 [MPa]	X/Y_SM	249	248	247	241	235	225	215	205	195	185	175
	X_HDM	-	172	160	160	159	158	158	157	157	156	154
	Y_HDM	-	180	181	181	176	172	169	166	165	164	163
Cut S1 [MPa]	X/Y_SM	151	147	143	137	130	124	118	111	105	99	93
	X_HDM	-	80	90	94	94	93	92	91	90	90	87
	Y_HDM	-	62	72	75	75	75	74	74	74	73	72
Uncut S3 [MPa]	X/Y_SM	247	246	245	238	231	220	208	197	186	176	166
	X_HDM	-	160	158	161	160	158	158	158	158	158	157
	Y_HDM	-	183	185	184	179	174	170	167	166	166	164
Cut S3 [MPa]	X/Y_SM	11	12	13	11	10	8	6	5	5	3	2
	X_HDM	-	23	21	22	22	22	22	22	22	21	20
	Y_HDM	-	2	8	12	13	14	14	15	16	16	15

**Table A2:** S1 and S3 on the 6 h bridges from Simufact Additive and HDM.

	D [mm]	0.0	0.1	0.2	0.3	0.4	0.5	0.6	0.7	0.8	0.9	1.0
Uncut S1 [MPa]	X/Y_SM	238	237	237	235	232	227	222	212	203	192	182
	X_HDM	-	147	141	139	137	136	134	133	132	131	130
	Y_HDM	-	141	146	149	149	148	146	144	142	141	139
Cut S1 [MPa]	X/Y_SM	110	111	112	114	117	116	115	110	105	99	93
	X_HDM	-	50	51	52	52	52	52	51	51	51	52
	Y_HDM	-	69	72	73	72	71	70	69	68	68	67
Uncut S3 [MPa]	X/Y_SM	261	260	258	254	251	243	235	223	210	197	184
	X_HDM	-	136	134	135	135	135	135	134	133	133	132
	Y_HDM	-	144	148	151	151	150	149	147	146	145	144
Cut S3 [MPa]	X/Y_SM	−5	0	6	13	20	22	24	24	23	20	18
	X_HDM	-	8	10	12	13	13	14	14	14	15	15
	Y_HDM	-	3	9	13	15	17	17	18	18	19	19

**Table A3:** ΔS3 on the 0 h bridges from simulations and HDM.

	D [mm]	0.0	0.1	0.2	0.3	0.4	0.5	0.6	0.7	0.8	0.9	1.0
ΔS3 (Uncut S3 − Cut S3) [MPa]	ΔX/Y_SM	236	234	232	227	221	212	203	192	182	173	164
	ΔX_HDM	-	137	137	139	138	136	136	136	136	137	137
	ΔX_ANS	196	189	182	175	168	161	154	148	141	135	129
	ΔY_HDM	-	181	177	173	166	160	156	152	150	150	149
	ΔY_ANS	244	235	226	217	209	200	192	184	176	168	160

**Table A4:** ΔS3 on the 6 h bridges from simulations and HDM.

	D [mm]	0.0	0.1	0.2	0.3	0.4	0.5	0.6	0.7	0.8	0.9	1.0
ΔS3 (Uncut S3 − Cut S3) [MPa]	ΔX/Y_SM	267	259	252	241	230	220	210	199	188	177	166
	ΔX_HDM	-	128	124	124	122	121	121	120	119	118	117
	ΔX_ANS	173	166	160	154	148	142	136	130	124	119	113
	ΔY_HDM	-	141	138	138	135	133	131	129	127	126	125
	ΔY_ANS	105	101	97	93	90	86	82	79	76	72	69
